# Weighted single-step GWAS and gene network analysis reveal new candidate genes for semen traits in pigs

**DOI:** 10.1186/s12711-018-0412-z

**Published:** 2018-08-06

**Authors:** Daniele B. D. Marques, John W. M. Bastiaansen, Marleen L. W. J. Broekhuijse, Marcos S. Lopes, Egbert F. Knol, Barbara Harlizius, Simone E. F. Guimarães, Fabyano F. Silva, Paulo S. Lopes

**Affiliations:** 1 Animal Science Department, Universidade Federal de Viçosa, Viçosa, MG 36.570-000 Brazil; 2 Animal Breeding and Genomics, Wageningen University & Research, P.O. Box 338, 6700 AH Wageningen, The Netherlands; 3 Topigs Norsvin Research Center B.V., P.O. Box 43, 6640 AA Beuningen, The Netherlands; 4 Topigs Norsvin, Curitiba, PR 80.420-210 Brazil

## Abstract

**Background:**

In recent years, there has been increased interest in the study of the molecular processes that affect semen traits. In this study, our aim was to identify quantitative trait loci (QTL) regions associated with four semen traits (motility, progressive motility, number of sperm cells per ejaculate and total morphological defects) in two commercial pig lines (L1: Large White type and L2: Landrace type). Since the number of animals with both phenotypes and genotypes was relatively small in our dataset, we conducted a weighted single-step genome-wide association study, which also allows unequal variances for single nucleotide polymorphisms. In addition, our aim was also to identify candidate genes within QTL regions that explained the highest proportions of genetic variance. Subsequently, we performed gene network analyses to investigate the biological processes shared by genes that were identified for the same semen traits across lines.

**Results:**

We identified QTL regions that explained up to 10.8% of the genetic variance of the semen traits on 12 chromosomes in L1 and 11 chromosomes in L2. Sixteen QTL regions in L1 and six QTL regions in L2 were associated with two or more traits within the population. Candidate genes *SCN8A*, *PTGS2*, *PLA2G4A*, *DNAI2*, *IQCG* and LOC102167830 were identified in L1 and *NME5*, *AZIN2*, *SPATA7*, *METTL3* and *HPGDS* in L2. No regions overlapped between these two lines. However, the gene network analysis for progressive motility revealed two genes in L1 (*PLA2G4A* and *PTGS2*) and one gene in L2 (*HPGDS*) that were involved in two biological processes i.e. eicosanoid biosynthesis and arachidonic acid metabolism. *PTGS2* and *HPGDS* were also involved in the cyclooxygenase pathway.

**Conclusions:**

We identified several QTL regions associated with semen traits in two pig lines, which confirms the assumption of a complex genetic determinism for these traits. A large part of the genetic variance of the semen traits under study was explained by different genes in the two evaluated lines. Nevertheless, the gene network analysis revealed candidate genes that are involved in shared biological pathways that occur in mammalian testes, in both lines.

**Electronic supplementary material:**

The online version of this article (10.1186/s12711-018-0412-z) contains supplementary material, which is available to authorized users.

## Background

Artificial insemination (AI) pig industry focuses mainly on maximizing the number of insemination doses produced from each boar ejaculate. To achieve this goal, the ability of boars to produce high-quality semen (high motility and progressive motility with low levels of morphological defects) in sufficient quantity (large number of sperm cells per ejaculate) is decisive [1].

In recent years, with the fast advances in high-throughput genotyping and in molecular techniques in general, there is an increased interest in the study of the molecular processes and genetic mechanisms that affect semen traits. Genes and markers associated with pig semen traits have been described in the literature [2–8]. However, very few studies analyze large datasets to identify novel quantitative trait loci (QTL) and to provide a deeper knowledge of the genes that control boar semen traits. One reason for this is the genetic complexity of the process for the production and maturation of sperm cells. Mammalian spermatogenesis requires coordination among different genes and cell types (germ cells, Sertoli cells, and Leydig cells) [9] and occurs in the seminiferous tubules of the testes in three steps: mitotic phase, meiotic phase, and spermiogenesis [10]. In the first step (mitosis), spermatogonias produce primary spermatocytes, which enter the first stage of meiosis (meiosis I) and produce secondary spermatocytes. Then, the second step of meiosis (meiosis II) leads to the generation of haploid round spermatids. In the last phase, i.e. spermiogenesis, the spermatids undergo morphological transformations and acquire the spermatozoa shape. Then, the new pre-formed spermatozoa go through the epididymis to maturate and acquire motility [10]. Mutations and impaired expression of genes that control the whole process of spermatogenesis and sperm maturation can lead to problems in semen quality and fertility.

Genome-wide association studies (GWAS) are commonly used to identify single nucleotide polymorphisms (SNPs) that are associated with QTL with major effects [11]. The weighted single-step GWAS (WssGWAS), proposed by Wang et al. [12], is a method that allows estimation of SNP effects using genomic estimated breeding values (GEBV) from single-step genomic best linear unbiased prediction (ssGBLUP, [13]) based on all phenotyped, genotyped and pedigree-related animals. In addition, it allows unequal variances for SNPs, which results in improved precision of the estimation of SNP effects [12]. Therefore, when the number of animals with both phenotypes and genotypes is small and the traits are controlled by QTL with large effects, the WssGWAS may perform better than traditional GWAS methods. Recent studies have used this method for production, carcass and reproductive traits in livestock [14–23].

In a post-GWAS study, a gene network analysis can be performed for candidate genes related to QTL regions identified in GWAS. The gene network is used to investigate pathways and biological processes that are shared by these genes [24]. In addition, the biological information provided by these gene networks are helpful to understand genetic differences between populations for the same trait [25].

In this study, our aim was to identify QTL regions that are associated with four semen traits (motility, progressive motility, number of sperm cells per ejaculate and total morphological defects) in pigs. In addition, our aim was to identify candidate genes within those QTL regions that explained the highest proportions of genetic variance. To achieve our goal, we performed a WssGWAS in two commercial pig lines (L1: Large White type and L2: Landrace type), followed by gene network analyses to investigate the biological processes shared by genes that were identified for the same semen traits in these two lines.

## Methods

### Phenotypic, genotypic and pedigree data

Phenotypic data were available from two commercial pig lines, a Large White type line (L1) and a Landrace type line (L2), on ejaculate samples that were collected between January 2007 and October 2014. The evaluated traits were: (1) sperm motility (MOT), which is the proportion of moving sperm cells in an ejaculate; (2) sperm progressive motility (PROMOT), defined as the proportion of sperm cells that move in a straight line; (3) abnormal sperm cell number (ABN), which is the total number of sperm cells with morphological abnormalities; and (4) the total number of sperm cells in the ejaculate (Ncells per 10^6^ sperm cells). MOT and PROMOT were evaluated using the UltiMateTM CASA system (Hamilton Thorne Inc., Beverly, MA, USA). Ncells was calculated as the product of the semen volume (mL) and concentration (10^6^ mL^−1^, measured by the CASA system). The values of this measure were not normally distributed, and thus log-transformed before further analyses (lnNcells). Ejaculates evaluated for ABN were analyzed microscopically at a 1000× magnification by a trained technician with a phase contrast microscope and a thermal plate (BH-2, Olympus, Tokyo, Japan), counting 100 sperm cells per assessment. All semen traits were assessed on the day of semen collection using fresh semen.

The phenotypic data for MOT, PROMOT and lnNcells consisted of 43,455 ejaculates for L1 (866 boars) and 39,161 ejaculates for L2 (900 boars). For ABN, the phenotypic data consisted of 13,366 ejaculates for L1 (849 boars) and 9853 ejaculates for L2 (886 boars). The average number of ejaculates per boar (with standard deviations in parenthesis) for MOT, PROMOT and lnNcells were 50.18 (38.12) for L1 and 43.51 (36.37) for L2. For ABN, the average number of ejaculates per boar were 15.74 (11.19) for L1 and 11.12 (8.99) for L2. Number of boars with phenotypic data, mean, standard deviation, minimum and maximum values of semen traits in L1 and L2 are in Table 1.

**Table 1:** Descriptive statistics of semen traits

	Number of boars^a^	Mean^b^	SD^c^	Min^d^	Max^e^
MOT^f^					
L1 ^g^	866	86.5	7.1	10	100
L2	900	87.1	6.5	14	100
PROMOT					
L1	866	78.6	8.3	0	100
L2	900	77.4	7.9	0	100
lnN_cells_					
L1	866	25.0	0.4	24.0	26.4
L2	900	24.9	0.4	22.5	26.4
ABN					
L1	849	19.3	14.8	1	98
L2	886	14.4	12.6	1	99

^a^Number of boars with phenotypic data

^b^Mean values of semen traits for each pig line

^c^Standard deviation

^d^Minimum trait value of semen traits

^e^Maximum trait value of semen traits

^f^Semen traits: MOT: sperm motility; PROMOT: sperm progressive motility; lnN_cells_: number of sperm cells per ejaculate; ABN: total morphological abnormalities

^g^Pig lines: L1 = Large White and L2 = Landrace

Genotyping data for 3737 animals (856 males and 2881 females) from L1 and 3307 animals (953 males and 2354 females) from L2 were available. A majority of the animals (2718 for L1 and 2394 for L2) were genotyped using the Illumina PorcineSNP60 BeadChip (Illumina, Inc., San Diego, CA) but part of the animals from L1 (n = 1019) and L2 (n = 913) were genotyped using the (Illumina, Inc.) GeneSeek Custom 80 K SNP chip (GeneSeek Inc., Lincoln, NE). Quality control was performed by excluding SNPs that had an unknown position on the pig genome build 10.2 [26], that were located on sex chromosomes, that had a call rate lower than 0.95 or a minor allele frequency lower than 0.01, or that were in strong deviation from Hardy–Weinberg equilibrium (χ^2^ > 600). Animals for which the frequency of missing genotypes was higher than 0.05 were also excluded. After quality control, missing genotypes from animals genotyped with the SNP60 BeadChip were imputed with the software Beagle version 3.3.2 [27] to the set of SNPs on the SNP60 BeadChip that passed quality control. In addition, genotypes from the animals genotyped with the GeneSeek Custom 80 K SNP chip were imputed to the set of SNPs on the SNP60 BeadChip that passed quality control. After quality control, 39,945 and 41,478 SNPs remained for L1 and L2, respectively, and were used for the GWAS.

The complete pedigree included 8352 animals for L1 and 8271 animals for L2. The total number of animals that remained after pedigree pruning was 6724 for L1 and 6502 for L2. Most animals had either phenotypic or genotypic data. The number of animals with both phenotypes and genotypes was 349 for L1 and 446 for L2.

### Statistical analyses

The weighted ssGBLUP analysis was conducted within line using the BLUPF90 software family [28] adapted for genomic analyses [29]. First, variance components were estimated using AIREMLF90, which were then used in BLUPF90 to predict GEBV. SNP effects were then calculated using postGSf90 software.

The single-trait animal model for ssGBLUP was as follows:

$${\mathbf{y}} = {\mathbf{X}}\varvec{\upbeta} + {\mathbf{Za}} + {\mathbf{Wp}} + {\varvec{\upvarepsilon}},$$

where ${\mathbf{y}}$ is the vector of phenotypic observations; ${\varvec{\upbeta}}$ is the vector of fixed effects (combined effects of AI station, year and month of semen collection; the laboratory where the samples were analyzed and the covariates of interval between two subsequent semen collections in days and age of the boar at the time of collection in months); ${\mathbf{a}}$ is the vector of random additive genetic effects; ${\mathbf{p}}$ is the vector of random permanent environmental effects; ${\varvec{\upvarepsilon}}$ is the vector of random residuals; and ${\mathbf{X}}$, ${\mathbf{Z}}$ and ${\mathbf{W}}$ are the incidence matrices of ${\varvec{\upbeta}}$, ${\mathbf{a}}$ and ${\mathbf{p}}$, respectively.

It was assumed that ${\mathbf{a}}\sim{\text{N}}\left( {0,{\mathbf{H}}\sigma_{a}^{2} } \right)$, ${\mathbf{p}}\sim{\text{N}}\left( {0,{\mathbf{I}}\sigma_{p}^{2} } \right)$ and ${\varvec{\upvarepsilon}}\sim{\text{N}}\left( {0,{\mathbf{I}}\sigma_{e}^{2} } \right),$ where $\sigma_{a}^{2}$, $\sigma_{p}^{2}$ and $\sigma_{e}^{2}$ are the additive genetic, permanent environmental and residual variances, respectively; ${\mathbf{H}}$ is the matrix that combines pedigree and genomic information [13], and ${\mathbf{I}}$ is an identity matrix. The inverse of ${\mathbf{H}}$ needed for mixed model equations is given by:

$${\mathbf{H}}^{ - 1} = {\mathbf{A}}^{ - 1} + \left[ {\begin{array}{*{20}c} 0 &\quad 0 \\ 0 &\quad {{\mathbf{G}}^{ - 1 } - {\mathbf{A}}_{22}^{ - 1} } \\ \end{array} } \right],$$

where ${\mathbf{A}}$ is the numerator relationship matrix based on pedigree for all animals; ${\mathbf{A}}_{22}$ is the numerator relationship matrix for genotyped animals; and ${\mathbf{G}}$ is the genomic relationship matrix [30]. This matrix was obtained as follows:

$${\mathbf{G}} = \frac{{{\mathbf{ZDZ}}^{{\prime }} }}{{\sum_{{{\text{i}} = 1}}^{\text{M}} 2{\text{p}}_{\text{i}} \left( {1 - {\text{p}}_{\text{i}} } \right)}},$$

where ${\mathbf{Z}}$ is a matrix of gene content adjusted for allele frequencies (0, 1 or 2 for *aa*, *Aa* and *AA*, respectively); ${\mathbf{D}}$ is a diagonal matrix of weights for SNP variances (initially ${\mathbf{D}} = {\mathbf{I}}$); ${\text{M}}$ is the number of SNPs, and ${\text{p}}_{\text{i}}$ is the minor allele frequency of ${\text{i}}$-th SNP.

Estimates of SNP effects and weights for WssGWAS were obtained according to Wang et al. [12] by the following steps:

1. In the first iteration (${\text{t}} = 1$): ${\mathbf{D}} = {\mathbf{I}}$; ${\mathbf{G}}_{{\left( {\text{t}} \right)}} = {\mathbf{D}}_{{\left( {\mathbf{t}} \right)}} {\mathbf{Z}}^{{\prime }}\uplambda$, where $\uplambda = \frac{1}{{\sum_{i = 1}^{M} 2p_{i} \left( {1 - p_{i} } \right)}}$ [30];
2. GEBV were calculated for the entire dataset using ssGBLUP;
3. GEBV were converted to estimates of SNP effects (${\hat{\mathbf{u}}}$): ${\hat{\mathbf{u}}}_{{\left( {\text{t}} \right)}} =\uplambda{\mathbf{D}}_{{\left( {\text{t}} \right)}} {\mathbf{Z}}^{{\prime }} {\mathbf{G}}_{{\left( {\text{t}} \right)}}^{ - 1} \hat{\varvec{a}}_{g}$, where $\hat{\varvec{a}}_{g}$ is the GEBV of animals that were also genotyped;
4. The weight for each SNP to be used in the next iteration was calculated as: $d_{{{\text{i}}\left( {{\text{t}} + 1} \right)}} = \hat{u}_{{{\text{i}}\left( {\text{t}} \right)}}^{2} 2p_{\text{i}} (1 - p_{\text{i}} )$, where ${\text{i}}$ is the ${\text{i}}$-th SNP;
5. The SNP weights were normalized to keep the total genetic variance constant:
  $${\mathbf{D}}_{{\left( {{\text{t}} + 1} \right)}} = \frac{{{\text{tr}}\left( {{\mathbf{D}}_{\left( 1 \right)} } \right)}}{{{\text{tr}}\left( {{\mathbf{D}}_{{\left( {{\text{t}} + 1} \right)}} } \right)}}{\mathbf{D}}_{{\left( {{\text{t}} + 1} \right)}} ;$$
6. $${\mathbf{G}}_{{\left( {{\text{t}} + 1} \right)}} = {\mathbf{ZD}}_{{\left( {{\text{t}} + 1} \right)}} {\mathbf{Z}}^{{\prime }}\uplambda$$ was calculated;
7. ${\text{t }} = {\text{t }} + 1$ and loop to step 2.

This procedure was run for three iterations based on the realized accuracies of GEBV according to Legarra et al. [31] and performed by Wang et al. [14]. At each iteration, the weights for SNPs were updated (steps 4 and 5), used to construct the ${\mathbf{G}}$ matrices (step 6), update the GEBV (step 2) and, consequently, the estimated SNP effects (step 3). The percentage of genetic variance explained by the ${\text{i}}$-th set of consecutive SNPs (${\text{i}}$-th SNP window) was calculated as described by Wang et al. [14] as:

$$\frac{{Var \left( {a_{\text{i}} } \right)}}{{\sigma_{a}^{2} }} \times 100\% = \frac{{Var\left( {\sum_{j = 1}^{x} {\mathbf{Z}}_{j} \hat{u}_{\text{j}} } \right)}}{{\sigma_{a}^{2} }} \times 100\% ,$$

where $a_{\text{i}}$ is the genetic value of the ${\text{i}}$-th SNP window that consists of a region of consecutive SNPs located within 0.4 Mb, which is the average haplotype block size in commercial pig lines [32], including the lines considered in the present study; $\sigma_{a}^{2}$ is the total additive genetic variance; ${\mathbf{Z}}_{j}$ is the vector of gene content of the $j$-th SNP for all individuals and $\hat{u}_{j}$ is the effect of the $j$-th SNP within the ${\text{i}}$-th window. Manhattan plots showing these windows were created using the R software [33].

### Selection of relevant SNP windows, search for candidate genes, and gene network analyses

The selection of relevant SNP windows and the search for genes within these QTL regions were performed in three steps. First, for the four traits (MOT, PROMOT, lnNcells and ABN), the SNP windows that explained 1% or more of the total genetic variance based on the WssGWAS were selected within each line (L1 and L2). The threshold of 1% was chosen based on the literature [19, 20, 34] and on the expected contribution of SNP windows [35]. For example, assuming an equal contribution of all windows (on average, we had 4223 and 4229 windows for each trait in L1 and L2, respectively), the expected proportion of genetic variance explained by each window was 0.02% for both lines (100/4223 for L1 and 100/4229 for L2). The threshold of 1% used in the present study is equal to 50 times the expected variance (0.02% × 50 = 1%) and considered a suitable threshold for our purposes. Then, after selecting the windows that explained more than 1% of the genetic variance, a search for overlapping windows for two or more traits of the same line was performed. Windows were considered to overlap if their midpoints were less than 0.4 Mb apart. Second, the three most important windows (that explained the highest proportion of genetic variance) for each trait and the 0.4 Mb region on either side of these windows midpoints were also identified. Third, based on the windows selected in the first step (> 1% of variance explained), overlapping windows for the same traits, but across lines, were investigated (also considering a maximum distance of 0.4 Mb between midpoints).

Based on the start and end positions of each identified and selected QTL region in the third step, we identified the genes in these QTL regions based on the Gene database for *Sus scrofa* available at “National Center for Biotechnology Information” (NCBI, [36]). For all the identified genes, we manually searched the literature if they had a previously identified relationship with the traits under study. In addition, based on human genes with the same description, we carried out four gene network analyses that described the biological processes and relations between the L1 and L2 sets of genes that were identified for the same traits by using the ClueGO and CluePedia Cytoscape plug-ins [37, 38]. The ClueGO plug-in combines Gene Ontology (GO) terms and KEGG/BioCarta pathways and develops a GO/pathway network. It also calculates enrichment and depletion tests for groups of genes based on the hypergeometric distribution and corrects the P-values for multiple testing [37]. Using the CluePedia plug-in, new associations between genes can be discovered with enrichments and added to the ClueGO pathways [38].

## Results

Estimates of variance components for all semen traits in both lines are in Additional file 1: Table S1. Low to moderate heritabilities ranging from 0.10 to 0.31 were estimated (Table 2), with higher estimates in L1 than L2 for MOT, PROMOT and ABN.

**Table 2:** Estimates (standard error) of heritabilities for the evaluated semen traits in two lines

Semen traits^a^	Pig lines^b^	
	L1	L2
MOT	0.21 (0.05)	0.12 (0.04)
PROMOT	0.31 (0.05)	0.12 (0.03)
lnNcells	0.10 (0.03)	0.13 (0.03)
ABN	0.22 (0.05)	0.14 (0.05)

^a^MOT: sperm motility; PROMOT: sperm progressive motility; lnNcells: number of sperm cells per ejaculate; ABN: total morphological abnormalities

^b^L1: Large White type and L2: Landrace type

**Fig. 1:**
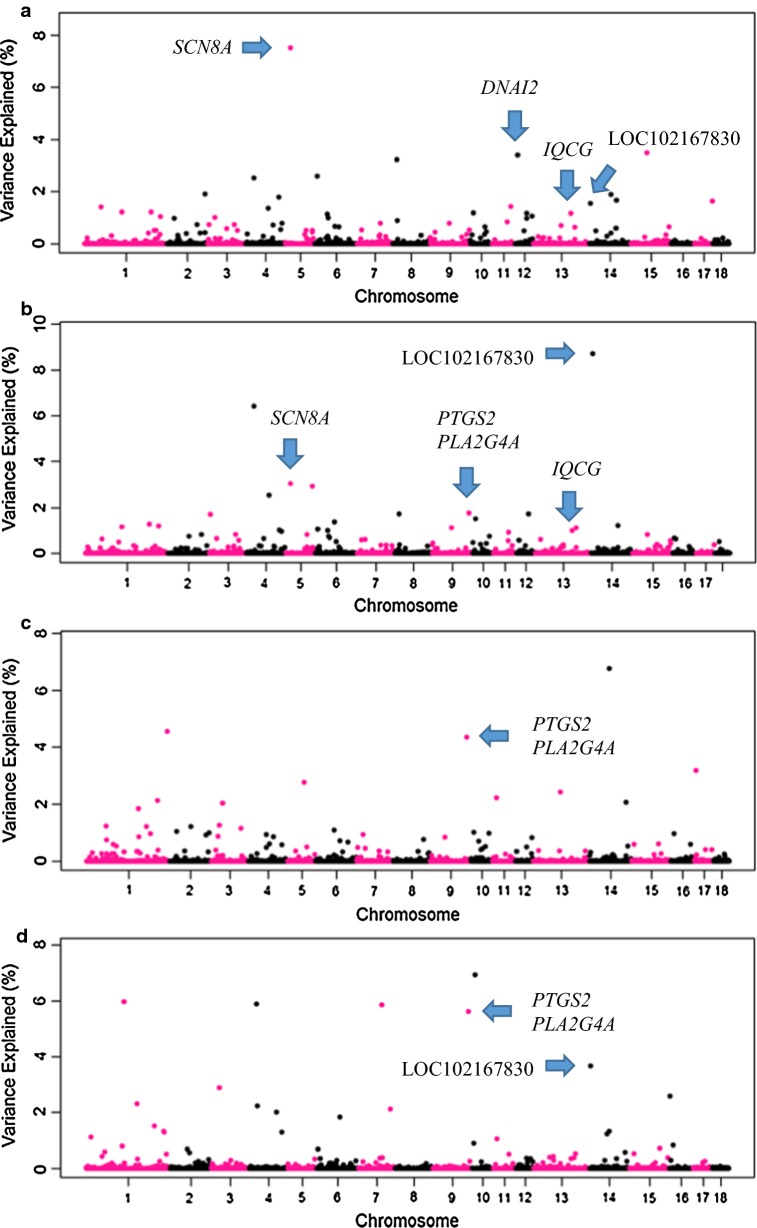
GWAS results of semen traits in the Large White type line (L1). **a** Sperm motility, **b** sperm progressive motility, **c** number of sperm cells per ejaculate, **d** total morphological abnormalities. Each dot represents one SNP window of 0.4 Mb. On the y-axis is the percentage of genetic variance explained by windows

**Fig. 2:**
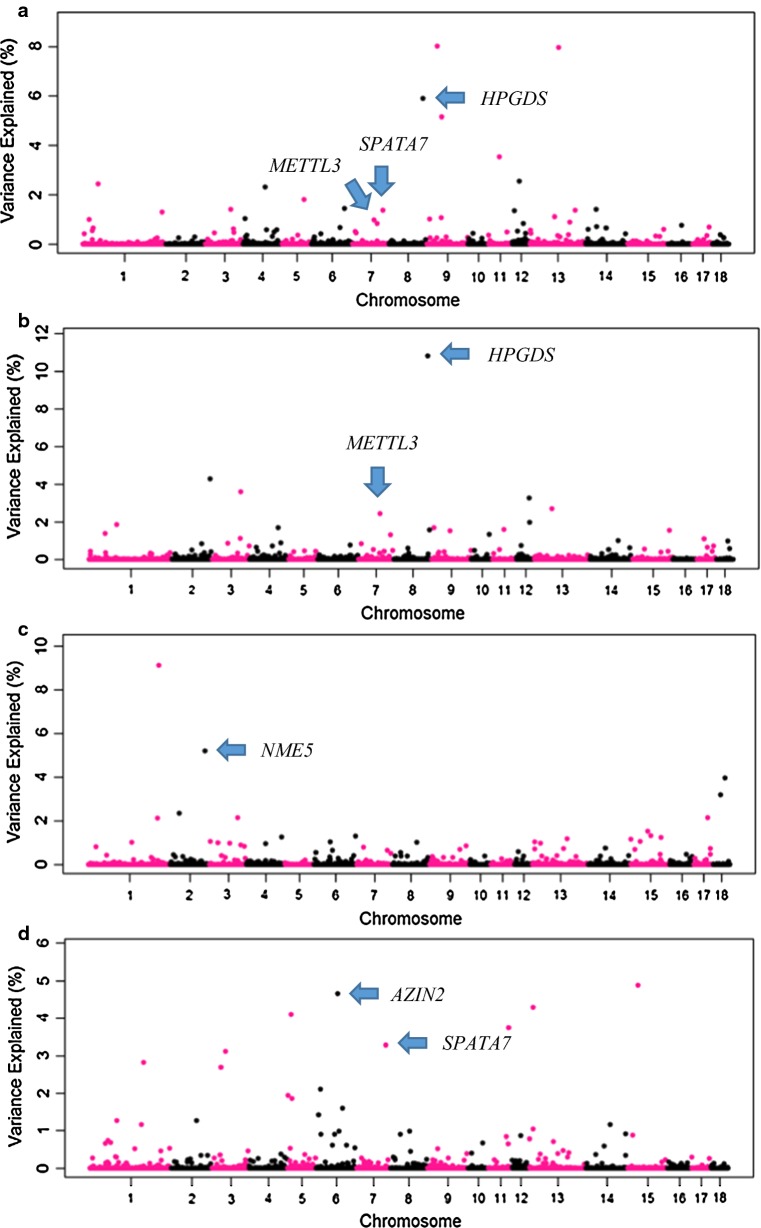
GWAS results of semen traits in the Landrace type line (L2). **a** Sperm motility, **b** sperm progressive motility, **c** number of sperm cells per ejaculate, **d** total morphological abnormalities. Each dot represents one SNP window of 0.4 Mb. On the y-axis is the percentage of genetic variance explained by windows

For L1, the three most important windows for MOT, PROMOT, lnNcells and ABN explained 14.4, 18.2, 15.67 and 18.8% of the genetic variance, respectively (Fig. 1) and Additional file 2: Table S2. For L2, the three most important windows explained 21.9, 18.7, 18.3 and 13.8% of the genetic variance of each trait, respectively (Fig. 2) and Additional file 3: Table S3. For L1, 20 relevant QTL regions (single and overlapping) were found on SSC1 (SSC for *Sus scrofa* chromosome), 3, 4, 5, 6, 8, 9, 10, 12, 13, 14, and 15 (Table 3). For L2, 16 relevant QTL regions were located on SSC1, 2, 3, 6, 7, 8, 9, 11, 13, 15 and 18 (Table 4). For L1, 110 genes located in the relevant QTL regions were identified, of which six genes were described to be related to MOT, PROMOT, lnNcells and ABN in the literature (Table 3). For L2, 106 genes were found in the detected relevant regions, of which five genes were related to MOT, PROMOT, lnNcells and ABN based on literature (Table 4).

**Table 3:** Individual and overlapping QTL regions for semen traits in L1

Chr^a^	QTL region (Mb)^b^	Nb SNP^c^	Var (%)^d^	Var (%)	Var (%)	Var (%)	Candidate gene^g^
			MOT^e^	PROMOT	lnNcells	ABN	
1	135.51–136.31	17	1.2	1.2	-^f^	6.0	-^h^
1	255.48–256.28	17	1.2	–	–	1.5	–
1	290.90–291.84	25	1.0	–	–	1.3	–
1	305.18–305.98	23	–	–	4.6	–	–
3	28.53–29.33	18	1.0	–	–	2.9	–
4	28.25–29.05	12	2.5	6.4	–	5.9	–
4	84.90–85.73	21	1.4	2.5	–	–	–
4	123.12–124.20	30	1.8	1.0	–	1.3	–
5	17.61–18.47	26	7.5	3.0	–	–	*SCN8A*
6	8.24–9.13	20	2.6	1.1	–	–	–
8	16.06–16.86	20	3.2	1.7	–	–	–
9	139.53–140.63	23	–	1.8	4.3	5.6	*PTGS2*, *PLA2G4A*
10	10.58–11.45	23	1.2	1.5	1.0	6.9	–
12	6.23–7.03	32	3.4	–	–	–	*DNAI2*
12	40.76–41.56	17	1.2	1.7	–	–	
13	143.61–144.69	13	1.2	1.0	–	–	*IQCG*
14	4.13–5.22	19	1.5	8.7	–	3.7	LOC102167830
14	72.83–73.63	16	–	–	6.8	–	–
14	99.70–100.51	25	1.7	1.2	–	–	–
15	61.93–62.73	15	3.5	–	–	–	–

^a^Chromosome

^b^Position of QTL region

^c^Number of SNPs within the QTL region

^d^Percentage of genetic variance explained by the QTL region

^e^Semen traits: MOT: sperm motility; PROMOT: sperm progressive motility; lnNcells: number of sperm cells per ejaculate; ABN: total morphological abnormalities

^f^The percentage of genetic variance explained by the QTL region is < 1%. When the variance is reported for more than one trait, the QTL region is overlapping across traits

^g^Best candidate gene(s) in the region

^h^No candidate genes associated with the trait

**Table 4:** Individual and overlapping QTL regions for semen traits in L2

Chr^a^	QTL region (Mb)^b^	Nb SNP^c^	Var (%)^d^	Var (%)	Var (%)	Var (%)	Candidate gene^g^
			MOT^e^	PROMOT	lnNcells	ABN	
1	270.94–271.74	10	-^f^	–	9.1	–	-^h^
1	55.61–56.47	26	2.4	1.4	–	–	–
2	145.69–146.49	16	–	–	5.2	–	*NME5*
2	154.03–154.83	23	–	4.3	–	–	–
3	110.29–111.09	19	–	3.6	–	–	–
6	83.32–84.12	12	–	–	–	4.7	*AZIN2*
7	116.37–117.28	25	1.4	–	–	3.3	*SPATA7*
7	82.56–83.36	10	1.0	2.4	–	–	*METTL3*
8	133.90–134.94	20	5.9	10.8	–	–	*HPGDS*
9	36.46–37.26	12	8.0	–	–	–	–
9	9.32–10.31	19	1.0	1.7	–	–	–
11	41.05–41.85	11	3.5	1.6	–	–	–
13	11.35–12.15	19	–	–	–	4.3	–
13	107.48–108.28	10	8.0	–	–	–	–
15	37.17–37.97	20	–	–	–	4.9	–
18	42.80–43.60	19	–	–	4.0	–	–

^a^Chromosome

^b^Position of QTL region

^c^Number of SNPs within the QTL region

^d^Percentage of genetic variance explained by the QTL region

^e^Semen traits: MOT: sperm motility; PROMOT: sperm progressive motility; lnNcells: number of sperm cells per ejaculate; ABN: total morphological abnormalities

^f^The percentage of genetic variance explained by the QTL region is < 1%. When the variance is reported for more than one trait, the QTL region is overlapping across traits

^g^Best candidate gene(s) in the region

^h^No candidate genes associated with the trait

**Fig. 3:**
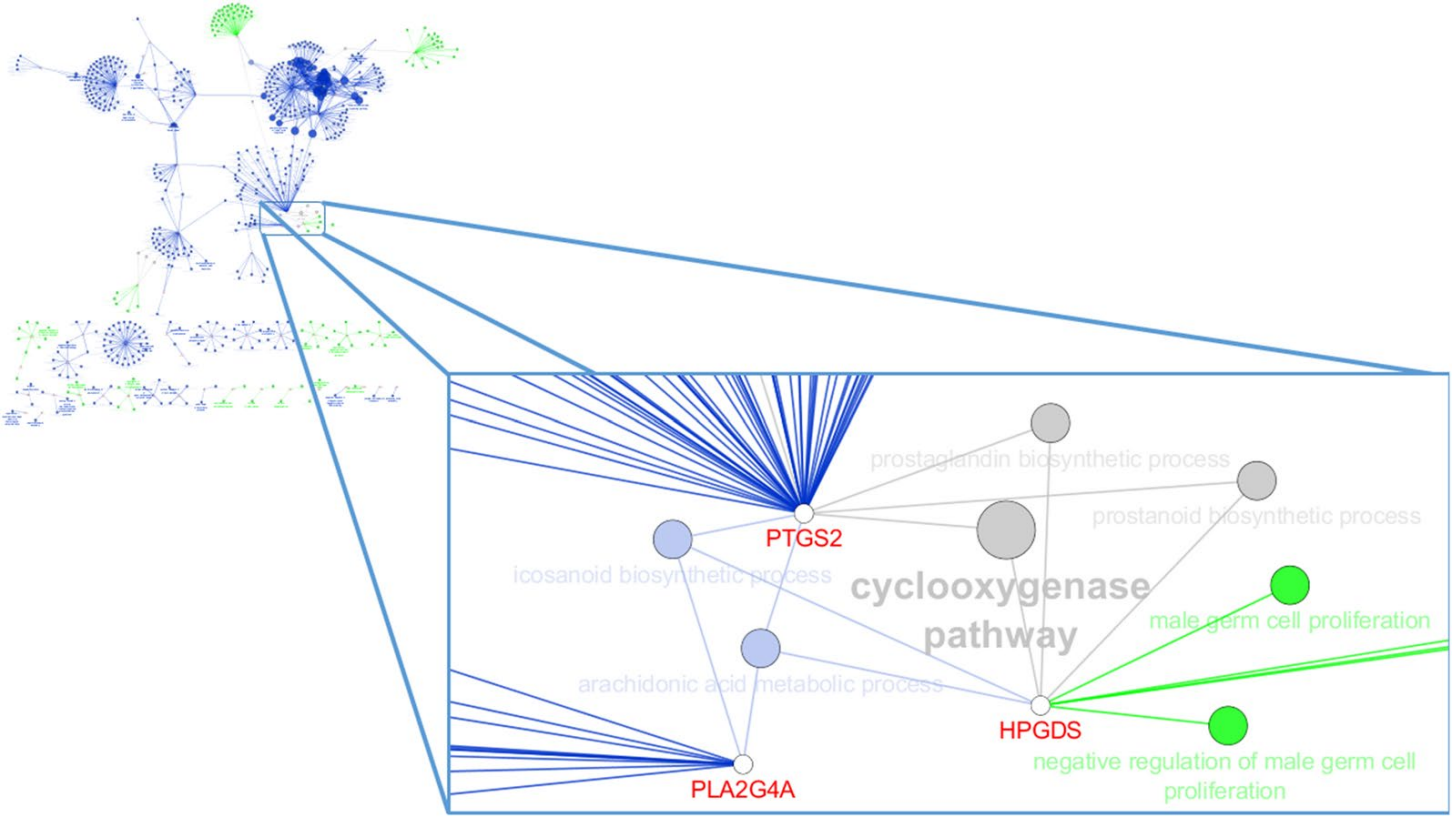
Gene network of biological processes for progressive motility. Complete network and important shared pathways (with zoom) are shown. Blue color indicates pathways for the Large White type line (L1) and green color indicates pathways for the Landrace type line (L2). Processes shared by *PTGS2* and *PLA2G4A* genes (L1) and *HPGDS* gene (L2) are connected by blue nodes. Processes shared by *PTGS2* and *HPGDS* are connected by grey nodes. Green dots are biological processes for *HPGDS*

We found no overlapping QTL regions between the two lines for the same traits in this study. Nevertheless, the gene network analysis for PROMOT revealed two genes in L1 (*PLA2G4A* and *PTGS2*) and one gene in L2 (*HPGDS*) that shared the biological processes of eicosanoid biosynthesis and arachidonic acid metabolism. The genes *PTGS2* in L1 and *HPGDS* in L2 were also found to share the biological process of cyclooxygenase pathway (Fig. 3).

## Discussion

In the past years, interest in investigating genomic regions that control boar semen traits has increased due to advances in molecular and genotyping techniques, statistical methods, and the ease of GWAS application. According to Wang et al. [12], two of the most used GWAS methods are (1) single-SNP GWAS, which fits each SNP separately as a fixed effect in a model that accounts for population stratification; and (2) Bayesian methods, which analyze all SNPs at the same time. Nonetheless, when the number of animals that are both phenotyped and genotyped is relatively small, the application of single-SNP GWAS and Bayesian methods is limited, since the calculation of pseudo-phenotypes (i.e. deregressed breeding values [39]) is necessary [14]. An alternative method is single-step GWAS (ssGWAS), which was proposed by Wang et al. [12] and converts the GEBV of genotyped animals obtained by ssGBLUP into SNP effects. However, the ssGWAS model, which assumes equal variance for all marker effects, may be limited when traits are affected by large QTL [12, 40]. To overcome this limitation, Wang et al. [12] proposed the WssGWAS approach, in which SNP effects are weighted according to their importance for the trait of interest, which improves QTL detection [12].

To perform the WssGWAS, first, the ${\mathbf{H}}^{ - 1}$ matrix is calculated, by combining all known pedigree and genotype information, and then used in the ssGBLUP procedure to estimate GEBV for all animals. Then, the GEBV of the genotyped animals are used to estimate effects for the SNPs. Finally, SNP effects are used to calculate the percentage of genetic variance that is explained by sets of consecutive SNPs (SNP windows). In this approach, the SNP effects are not directly estimated from the model and there are no measures of uncertainty for the statistical tests. However, the WssGWAS provides information on the most important SNP windows, based on the proportion of explained genetic variance, which is a general concept that is widely accepted in QTL detection analyses [12–14] but does not allow for a formal testing of significance. In this study, we chose the WssGWAS approach because: (1) it can integrate all phenotypic, genotypic and pedigree data simultaneously, thus avoiding the need to calculate pseudo-phenotypes for genotyped animals to incorporate all phenotypic information; (2) it allows the use of different weights for SNPs according to their importance, which is a deviation from the non-realistic GBLUP assumption of the infinitesimal model and improves the precision of estimates of SNP effects; and (3) it provides the possibility to work with SNP windows, since a window of consecutive SNPs in the GWAS may be more successful in finding QTL regions compared to individual SNP analysis because of linkage disequilibrium (LD). In general, analyses that consider the association of individual SNPs may overestimate the number of detected QTL [41].

In spite of the small number of animals that were both genotyped and phenotyped (349 for L1 and 446 for L2), all the information of the 3737 and 3307 genotyped animals, the 866 and 900 phenotyped boars (849 and 886 for ABN), and the 6724 and 6502 animals in the pedigree file, for L1 and L2, respectively, were used to calculate GEBV, and consequently, to estimate SNP effects in the WssGWAS. Obtaining a large dataset of both genotyped and phenotyped animals is unlikely or difficult for semen traits because phenotypes can be recorded only for animals that are used for AI. The number of males and females genotyped in this study also shows the relatively small number of boars, 856 and 953 for L1 and L2, respectively, compared to the number of females, 2881 and 2354. In this context, the advantage of WssGWAS is to supply additional information on relatives without genotypes in order to improve the statistical power of QTL detection [12].

In our previous study [7], a classical GWAS (single-SNP GWAS) was performed for sperm motility in a subset of the L1 and L2 populations that were used in the present study: 645 and 1886 phenotyped and genotyped animals for L1, respectively, and 760 and 1972 animals for L2. Due to the small number of animals that were both genotyped and phenotyped, deregressed breeding values [39] were calculated and included as response variables in the GWAS model, a polygenic effect was added in the model to account for population structure, and a genome-wide false discovery rate (FDR) was applied (q-values) to avoid false positives due to multiple testing. The results showed no SNPs with a significant association (q-value ≤ 0.05) with sperm motility for L1 but six SNPs associated with the trait for L2 (SSC1, from 117.26 to 119.50 Mb). In this QTL region on SSC1, a novel candidate gene (*MTFMT*) that affects translation efficiency of proteins in sperm cells, was identified. The number of relevant QTL regions that was identified for MOT in the present study was greater than in [7], which indicates that the WssGWAS was more successful in detecting QTL. All the recommended steps for the GWAS method used were performed in each study and reliable candidate genes with biological meaning were found. In addition, Marques et al. [42] reported high genetic correlations between MOT, PROMOT and ABN, which corroborate our findings regarding the overlapping QTL regions that explained more than 1% of the genetic variance for these traits. Therefore, we believe that the QTL results of the present and previous studies should not be considered as false positives and we conclude that the single-SNP method with pseudo-phenotypes and the amount of data in the previous study were not able to identify some of the relevant QTL related to MOT. For L1, we identified overlapping QTL regions for MOT and other semen traits on SSC1 (Table 3), but they did not include the *MTFMT* gene described in our previous study [7], likely as a result of the larger number of animals used in the current study and the different statistical models used in the two studies, i.e. WssGWAS based on the ${\mathbf{H}}$ relationship matrix in the current study and single-SNP GWAS using the ${\mathbf{A}}$ relationship matrix and deregressed phenotypes in the previous study.

In the WssGWAS, we identified several relevant QTL regions associated with the traits under study, which confirms the assumption that these traits have a complex genetic determinism. In this study, the region used to search for candidate genes was not limited to the SNP window, but also included the upstream and downstream flanking regions. The use of a larger genomic region for the identification of genes is important because SNPs within a window may be in high LD with QTL in the surrounding area.

We detected several candidate genes for the semen traits in both L1 and L2 pig lines (Tables 3 and 4). For L1, the *dynein axonemal intermediate chain 2* (*DNAI2*) gene, located on SSC12, was considered the best candidate gene for MOT in the QTL region. The *DNAI2* gene encodes axonemal dyneins; the axoneme is a microtubular structure located in the center of all motile cilia and flagella, including sperm flagella [43]. Dyneins are large multisubunit ATPases that interact with microtubules to generate the driving force for flagellar motility [44]. Mutations in the human *DNAI2* are involved in defects of the axoneme [45].

For L1, some overlapping QTL regions were identified between MOT and PROMOT. On SSC5, we identified one candidate gene, i.e. *sodium voltage*-*gated channel alpha subunit 8* (*SCN8A*). Pinto et al. [46] described the expression of *SCN8A* in human sperm flagellum principal piece and showed that sodium channels contribute to the regulation of human sperm motility. Another candidate gene for both traits was identified on SSC13, i.e. *IQ motif containing G* (*IQCG*). Li et al. [10] showed that *Iqcg* knockout mice presented severe malformation and total immobility of their spermatozoa because of disorganized sperm flagellum axoneme. Harris et al. [47] reported that mice with mutations in the *Iqcg* gene presented spermiogenesis defects, with incomplete sperm tail formation.

We detected one overlapping QTL region in L1 for MOT, PROMOT and ABN on SSC14. This QTL region includes the gene *LOC102167830*, which is described as a *spermatogenesis associated (SPATA) protein 31E1*-*like*. *SPATA* genes form a large gene family that plays a very important role in testis development and spermatogenesis [48]. In humans, *SPATA31E1* is a subfamily of the *SPATA31* large gene family. In *Mus musculus*, only the *Spata31* gene has been described. Wu et al. [49] demonstrated that the protein encoded by this mouse gene is located in the acrosome of round and elongated spermatids and *Spata31* knockout mice showed disorganized testis morphology and aberrant spermatogenic cells in seminiferous tubules.

**Fig. 4:**
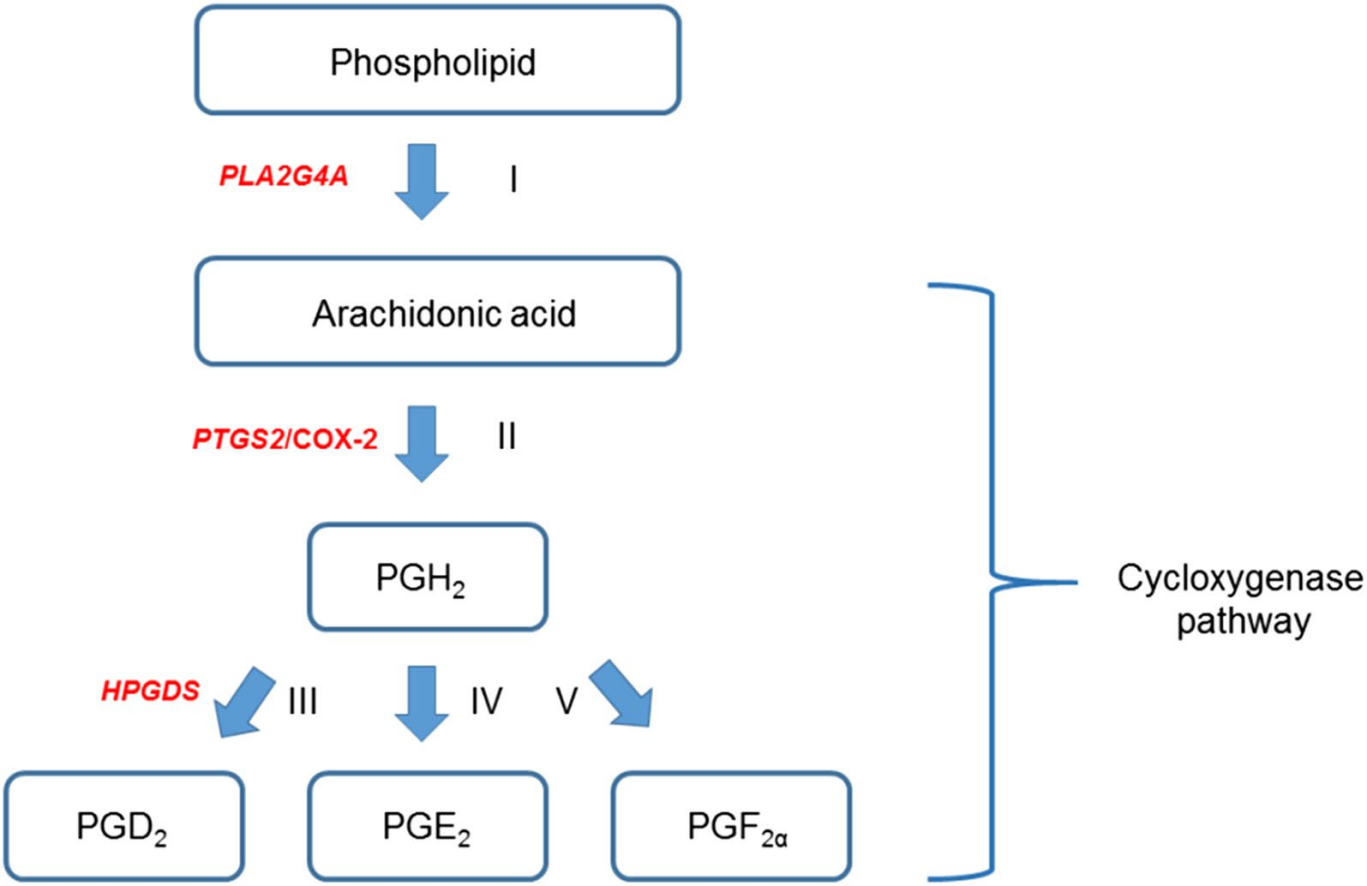
Graphic scheme of pathways shared by genes found in network analysis for progressive motility. Only part of the cyclooxygenase pathway is presented. *Cytosolic phospholipase A2 group IVA* (*PLA2G4A*) is involved in cleaving arachidonic acid from phospholipids, preferentially (I). Then, the free arachidonic acid is metabolized to produce eicosanoids (including prostaglandins) in the process known as cyclooxygenase pathway (II–V). The genes *prostaglandin*-*endoperoxide synthase 2* (*PTGS2*/*COX*-*2*, number II) and *hematopoietic prostaglandin D synthase* (*HPGDS*, number III) are involved in this pathway. The *COX*-*2* enzymes catalyze *prostaglandin H*_*2*_ (*PGH*_*2*_) synthesis from arachidonic acid (II), providing *PGH*_*2*_ for the action of *HPGDS* (III) and production of *prostaglandin D*_*2*_ (*PGD*_*2*_) in testes interstitial mast cells. *PGH*_*2*_ can also be converted into *prostaglandin E*_*2*_ (*PGE*_*2*_, number IV) and *prostaglandin F*_*2α*_ (*PGF*_*2α*_, number V)

The gene network analysis was very useful to investigate the shared biological processes between the candidate genes that were identified for the same traits between the two lines. For PROMOT, we found two genes in L1 (*PLA2G4A* and *PTGS2*) and one gene in L2 (*HPGDS*) that are involved in eicosanoid biosynthesis and arachidonic acid metabolism, of which *PTGS2* and *HPGDS* are also involved in the cyclooxygenase pathway (Figs. 3 and 4). Kaewmala et al. [50] described the presence of *PTGS2* (*COX*-*2*) protein in boar Leydig cells, spermatogonium and spermatids, which suggests that it may have a role in the spermatogenic process in pigs. They also showed that the levels of *COX*-*2* mRNA and enzyme tended to be higher in animals with low sperm motility, which indicates that it has a negative effect on boar sperm quality. Frungieri et al. [51] showed that the *COX-2* enzyme is abundant in the interstitial cells of male seminiferous tubules with impaired spermatogenesis. The *COX-2* enzyme provides a precursor for the action of *HPGDS* in testes interstitial mast cells (Fig. 4), producing *prostaglandin* *D*_2_ (*PGD*_*2*_), which regulates the function of Leydig cells [52]. Yamamoto et al. [53] and Saharkhiz et al. [54] showed that, after treatment with mast cells blockers, sperm motility increased in men. *HPGDS* is also involved in negative regulation of male germ cell proliferation (Fig. 3), which is linked to *PGD*_*2*_ production. Moniot et al. [55] identified the *PGD*_*2*_ pathway as one of the earliest signaling pathways involved in male germ cell differentiation in fetal testes. In the cyclooxygenase pathway, *PGH*_*2*_ can be converted into *prostaglandin* *E*_*2*_ (*PGE*_*2*_) or into *prostaglandin F*_*2α*_ (*PGF*_*2α*_) (Fig. 4, steps IV and V). Schlegel et al. [56] stated that seminal plasma is the richest natural source of prostaglandins, which are synthesized in the seminal vesicles. The authors showed that high concentrations of prostaglandins (especially *PGF*_*2α*_) in the human seminal fluid were associated with poor sperm motility. Rios et al. [57] demonstrated that low physiological levels of *PGE*_*2*_ and *PGF*_*2α*_ were able to increase and prolong human progressive sperm motility.

For L2, the *NME/NM23 family member 5* gene (*NME5*) on SSC2 was considered the best candidate for lnNcells (Table 4). According to Munier et al. [58], this gene is highly and specifically expressed in testis and the encoded protein is important for the initial stages of spermatogenesis (before meiosis I). Choi et al. [59] reported that, when expression of murine *Nm23*-*M5* (which shares 86% identity with its human homolog *NME5*) is reduced, the round and elongated spermatids in the testes become more sensitive to oxidative stress, leading to DNA damage and reduced cell numbers, showing that this gene is a critical factor for spermiogenesis.

The *antizyme inhibitor 2* gene (*AZIN2*) is located in a region on SSC6 that explained 4.7% of the genetic variance for ABN in L2 (Table 4). Lopez-Contreras [60] showed that *AZIN2* expression is critical for spermiogenesis in mice. Its expression starts in the testis at the beginning of spermiogenesis and its mRNA level increases in more differentiated spermatids. During spermiogenesis, round spermatids elongate, develop an acrosome in the sperm head, form a flagellum, and dispose of the excessive cytoplasm [10]. Therefore, if a mutation in *AZIN2* causes impaired spermiogenesis, it may lead to morphological defects in the spermatozoa.

The *methyltransferase like 3* gene (*METTL3*) is located on SSC7, in an overlapping QTL region for MOT and PROMOT in L2. According to Liu et al. [61], the METTL3 protein catalyzes the methylation and formation of N^6^-methyladenosine (m^6^A), which is the most prevalent and reversible RNA epigenetic modification in mammalian mRNA. Yang et al. [62] detected increased m^6^A contents in sperm RNA from patients with reduced sperm progressive motility, which was related to a higher expression of *METTL3*.

An overlapping QTL region for MOT and ABN was detected for L2 on SSC7 (Table 4), which includes the *spermatogenesis associated 7* gene (*SPATA7*). This gene was first identified in rat and human spermatocytes and may be involved in preparing chromatin for the initiation of meiotic recombination [63]. According to Ferguson et al. [64], meiotic recombination ties homologous chromosomes together and facilitates proper segregation of chromosomes during meiosis. Errors in the formation of crossovers can result in the production of aneuploid gametes. Sun et al. [65] reported a higher frequency of sperm aneuploidies for some chromosomes in men with sperm morphological defects compared to men with normal sperm concentrations. Thus, the *SPATA7* gene in this QTL region on SSC7 is considered the best candidate gene for MOT and ABN.

## Conclusions

We identified several QTL regions that are associated with semen traits in two pig lines using the weighted single-step GWAS, which allowed detection of QTL in spite of the small number of animals having both phenotypes and genotypes. A large part of the genetic variance of the semen traits was explained by different genes in the two lines but the gene network analysis revealed candidate genes for these two lines that are involved in shared biological pathways in the mammalian testes. These results can be used to search for causative mutations and for marker-assisted selection to enhance the production and quality of semen for a more efficient use of AI in pig breeding and production.

## Additional files

**Additional file 1: Table S1.** Variance components and standard errors (in parenthesis) for semen traits. The data provided represent the values of variance components (additive genetic, environmental and residual) and the respective standard errors for each semen trait and pig line.

**Additional file 2: Table S2.** The three SNP windows that explained the highest proportion of genetic variance of each semen trait in L1. The data provided represent the chromosome, average window position (in basepairs) and the percentage of variance explained by the three SNP windows that explained the highest proportion of genetic variance for each semen trait in L1.

**Additional file 3: Table S3.** Three SNP windows that explained the highest proportion of genetic variance of each semen trait in L2. The data provided represent the chromosome, average window position (in basepairs) and the percentage of variance explained by the three SNP windows that explained the highest proportion of genetic variance for each semen trait in L2.
